# Diatom-mediated food web functioning under ocean artificial upwelling

**DOI:** 10.1038/s41598-024-54345-w

**Published:** 2024-02-17

**Authors:** Silvan Urs Goldenberg, Carsten Spisla, Nicolás Sánchez, Jan Taucher, Kristian Spilling, Michael Sswat, Anna Fiesinger, Mar Fernández-Méndez, Bernd Krock, Helena Hauss, Jacqueline Haussmann, Ulf Riebesell

**Affiliations:** 1 Biological Oceanography, GEOMAR Helmholtz Centre for Ocean Research Kiel, Kiel, Germany; 2 Marine and Freshwater Solutions, Finnish Environment Institute, Helsinki, Finland; 3 Centre for Coastal Research, University of Agder, Kristiansand, Norway; 4 Department of Biology, University of Konstanz, Konstanz, Germany; 5 Alfred Wegener Institute Helmholtz Centre for Polar and Marine Research, Bremerhaven, Germany; 6 NORCE Norwegian Research Centre, Mekjarvik, Norway

**Keywords:** Marine biology, Biogeochemistry, Ecology

## Abstract

Enhancing ocean productivity by artificial upwelling is evaluated as a nature-based solution for food security and climate change mitigation. Fish production is intended through diatom-based plankton food webs as these are assumed to be short and efficient. However, our findings from mesocosm experiments on artificial upwelling in the oligotrophic ocean disagree with this classical food web model. Here, diatoms did not reduce trophic length and instead impaired the transfer of primary production to crustacean grazers and small pelagic fish. The diatom-driven decrease in trophic efficiency was likely mediated by changes in nutritional value for the copepod grazers. Whilst diatoms benefitted the availability of essential fatty acids, they also caused unfavorable elemental compositions via high carbon-to-nitrogen ratios (i.e. low protein content) to which the grazers were unable to adapt. This nutritional imbalance for grazers was most pronounced in systems optimized for CO_2_ uptake through carbon-to-nitrogen ratios well beyond Redfield. A simultaneous enhancement of fisheries production and carbon sequestration via artificial upwelling may thus be difficult to achieve given their opposing stoichiometric constraints. Our study suggest that food quality can be more critical than quantity to maximize food web productivity during shorter-term fertilization of the oligotrophic ocean.

## Introduction

Famine and malnutrition have been shaping human evolution and development over millennia^1,2^. Could farming of the open ocean with its vast space, energy and nutrients contribute to food security? Operations far beyond the continental shelfs may not only take some pressure off coastal and terrestrial ecosystems^3^, but also promote a healthy diet via seafood rich in protein, minerals and essential biomolecules^4,5^. Artificial upwelling has been proposed as a nature-based solution that pumps up nutrient-rich deep water to the sunlit surface to fuel productivity from primary producers to harvestable fish^6^. Its potential for negative emissions via long-term storage of the organically bound carbon is also being evaluated^7–11^. To provide these ecosystem services, however, artificial upwelling would rely on a specific functioning of the pelagic food web involving multiple species interactions.

The concept of artificial upwelling for fisheries production is founded on the classical food web model with diatoms as the base for efficient food webs^12^. These cosmopolitan primary producers are fast growing in nutrient-rich waters^13^ and their large size enables direct consumption by crustacean zooplankton such as copepods and hence a shortcut to fish^14,15^. Given that the majority of energy is lost with each trophic step—90%, as a rule of thumb—, a short food web bypassing the microbial loop would result in multi-fold higher trophic efficiency^16^. This diatom-paradigm is believed to sustain the world’s most productive fisheries in natural upwelling areas^17,18^.

In reality, the interaction between diatoms and their zooplankton grazers is more complex. While diatoms quickly convert new nutrients into biomass^19,20^, their quality as food for crustacean grazers and benefit for fisheries is debated^21–23^. Diatom palatability and nutritional value can be reduced by the characteristic silica shell via armament and digestive ballast^24,25^, toxic secondary metabolites^26,27^ and an imbalanced elemental and biochemical composition^28–30^. For artificial upwelling, such diatom traits may be particularly detrimental as here grazer communities originate from nutrient-poor waters and are habituated to a non-diatom diet^31,32^. Several generations may be required for the selection and proliferation of suitable species and phenotypes. This would mean months for copepods^33^. Any diatoms not utilized by grazers would eventually sink out of the surface^7,10,34,35^; energy and nutrients that are effectively lost to fisheries in deep oceanic waters.

Here we studied the role of diatoms in artificial upwelling food webs in the oligotrophic North Atlantic. We drew upon two large-scale mesocosm experiments that simulated upwelling of varying intensity and nutrient composition (Si:N). Diatoms bloomed in response to the fertilization and mediated biogeochemical processes including primary production^20,36,37^, grazing by heterotrophic protists^38^, suspended matter build-up^19^ and particle export^7,10^. Our current study expands this investigation to higher trophic levels. We studied trophic structure and efficiency with particular focus on food quality, crustacean zooplankton and small pelagic fish. Our research assesses the classical food web model and provides critical insights for the evaluation of artificial upwelling as a nature-based solution for food production and CO_2_ removal.

## Results

### Nutrient composition: experiment 1

**Figure 1:**
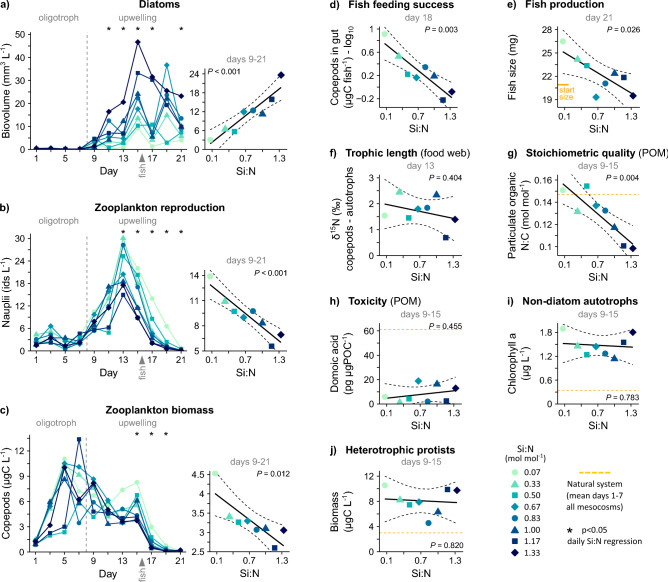
Food webs under varying diatom abundances, resulting from different Si:N during artificial upwelling. (**a**–**f**) Key trophic groups of the classical food web model. (**g**,**h**) Nutritional value of particulate organic matter (POM) as potential zooplankton food. (**i**,**j**) Alternative, non-diatom food options for zooplankton. Shown are temporal developments and averages employed in regressions (Table S1), with time intervals specified in grey and 95% confidence ranges via dashed lines. Plot (**a**) reproduced from Goldenberg et al.^19^.

Our first experiment simulated regular upwelling of nutrient-rich deep water to a surface plankton community. The amount of silicate was manipulated between the experimental units to obtain a gradient in silicate relative to nitrate (*Si:N*). This ratio varies in deep water across ocean regions and depth and limits diatom competitiveness at the sunlit surface (Si-based shells, Ref.^24^). As hypothesized, we found that Si availability during upwelling enhanced diatom blooms. Diatoms developed ~ 8 times larger populations under excess of Si compared to extreme Si deficiency (Fig. 1a). Phytoplankton productivity and biomass, the relative contribution of diatoms, and particle sizes increased along our Si:N gradient (presented in Goldenberg et al.^19^ and Ortiz et al.^37^). The conditions were set to test the bottom-up forcing diatoms exert on higher trophic levels.

Contrary to the classical food web model, we observed a reduced trophic efficiency under increased dominance of diatoms. This finding was based on crustacean zooplankton, primarily comprising herbivorous and omnivorous copepod species. Their larvae were half as abundant under higher compared to lower diatom presence (Fig. 1b), probably as a result of suppressed reproductive performance by adults. Shortly after, zooplankton biomass developed the same negative dependency on diatoms (Fig. 1c). Over the final 6 days, small pelagic fish preyed on and depleted the zooplankton populations. Their feeding success (Fig. 1d) and ultimately biomass growth (Fig. 1e) matched the availability in zooplankton prey. Fish production was hence reduced in upwelling communities high in Si and diatoms. The trophic inefficiency evidently occurred between phytoplankton and zooplankton, while the second trophic step to fish remained functional. Not surprising given these patterns in biomass, we found no evidence for a diatom-enabled shortening of the food web (Fig. 1f, Fig. S2b).

Next, we investigated possible pathways of impaired zooplankton grazing. Diatom-driven carbon overconsumption led to ~ 35% lower N content in food (Fig. 1g), implying reduced protein and nutritional value. The phycotoxin domoic acid was, in contrast, not related to the blooming diatoms and remained below levels of the oligotrophic system throughout the upwelling period (Fig. 1h, Fig. S3a,b). This was despite the presence of the diatom genus *Pseudo-nitzschia* of which some species have the potential to produce domoic acid^19,27^. The availability of non-diatom food also remained unaffected by diatoms. Considered were autotrophs (Fig. 1i, Fig. S3c) and heterotrophic protists (Fig. 1j) such as ciliates (Fig. S3d) and dinoflagellates (Fig. S3e). These alternative food options were however diluted by the over-proportional increase in lower-value diatoms (compare Fig. 1a with i and j).

### Upwelling intensity: experiment 2

**Figure 2:**
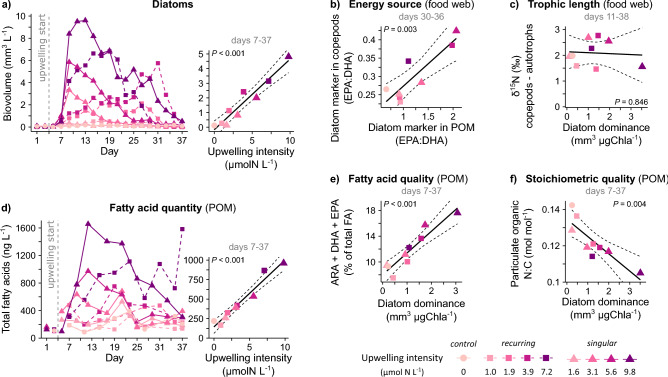
Food webs under varying diatom abundances, resulting from different intensities of artificial upwelling. New nutrients as drivers of (**a**) diatoms and (**d**) fatty acids in particulate organic matter (POM). (**b**,**c**) Trophic marker-based food web structure. (**e**,**f**) Nutritional value of potential zooplankton food. Shown are temporal developments and averages employed in regressions (Table S3), with time intervals specified in grey and 95% confidence ranges via dashed lines. Plot (**a**) reproduced from Spilling et al.^38^.

Our second experiment manipulated the amount of upwelled nutrients, at a constant nutrient composition. During real world application, this upwelling *intensity* may vary depending on nutrient concentrations at depth and pumping rates. We found that diatom blooms scaled linearly with intensity (Fig. 2a). This coincided with larger particle sizes and higher primary productivity (presented in Ortiz et al.^20,36^). While exact bloom dynamics depended on the *mode* of nutrient supply—in a single, large pulse (*singular*) or in regular, smaller pulses (*recurring*)— upwelling intensity still emerged as the main diatom driver (Fig. 2a). We now investigated how a dominance of diatoms (Fig. S4a) may influence key properties of the plankton food web.

According to our trophic marker analysis, at least some of the diatom productivity was channeled up to crustacean zooplankton (Fig. 2b, Fig. S5b). Nevertheless, as in the first experiment, diatoms did not shorten the food web (Fig. 2c, Fig. S5c). Clearly, while diatoms were no trophic dead-end, they were not able to provide a shortcut to zooplankton either. This indicates that heterotrophic protists remained important trophic intermediaries even under diatom dominance.

In terms of food quality for grazers, we observed contrasting effects of diatoms. On the one hand, blooming diatoms produced a substantial quantity of fatty acids that are of high value for food web functioning and human health^5,30^. Fatty acids increased by up to an order of magnitude (Fig. 2d) and their content in suspended biomass by 80% (Fig. S4b). Their composition also shifted towards those most essential for consumers, including a relative doubling in key long-chain fatty acids (ARA, DHA and EPA) (Fig. 2e, Fig. S4c), polyunsaturated fatty acids (PUFAs) (Fig. S4d) and in ω3/ω6 (Fig. S4e). On the other hand, food N content declined by up to 30% with increasing diatom dominance (Fig. 2f), like in the first experiment.

### Ecological stoichiometry

**Figure 3:**
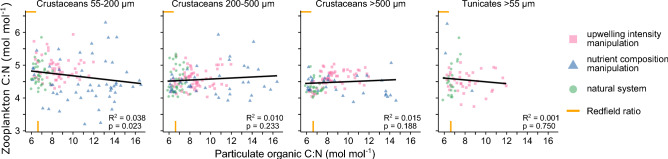
Stoichiometric constraints of grazers under artificial upwelling with varying levels of carbon overconsumption in phytoplankton**.** The body composition of grazers is compared to that of their potential food across all experiments, mesocosms and time points. Only herbivorous or omnivorous taxa are considered including copepods and cladocerans (crustaceans) and appendicularians and doliolids (tunicates). Statistics are based on linear mixed models (Table S4).

Diatom-driven changes in food stoichiometry towards higher carbon-to-nutrient ratios well beyond Redfield were omnipresent during artificial upwelling, both under varying upwelling intensity and nutrient composition. While our analysis focuses on nitrogen (e.g. in proteins) as the limiting nutrient locally^19,20^ and over much of the global ocean^39^, phosphorus (e.g. in RNA) behaved similarly (Fig. S6). Based on these findings, Goldenberg et al.^19^ hypothesized a stoichiometric imbalance during trophic transfer, as heterotrophs are thought to be less flexible in their elemental composition compared to autotrophs^40^. Here we show that zooplankton body composition did indeed not adjust to the elevated carbon-to-nutrient ratios in their food (Fig. 3). This stoichiometric homeostasis was consistent across all herbivores and omnivores, from small to large sizes and from crustacean to gelatinous taxa. As zooplankton maintained a body C:N ratio in a narrow range from 4 to 6, the stoichiometric imbalance between food and consumer often expanded well beyond the variability of the natural system.

## Discussion

Our study rates diatoms as a poor food source for copepod zooplankton and trophic efficiency during artificial upwelling. Diatoms were unable to shorten food webs and even reduced trophic transfer to small pelagic fish, the center of productive fisheries^17^. These findings from artificial upwelling in the oligotrophic ocean align with observations of zooplankton impairment in systems where diatoms are already more common naturally^21,22,25,26^.

Diatoms may have hindered copepod growth and reproduction in different ways. We collected support (or lack thereof) for potential mechanisms across this study and other articles published on these experiments (Table 1). Our diatoms comprised smaller taxa^19,20^ that fell within the theoretical food size range of the copepods^14^ and were similar in size to common prey of the natural, oligotrophic food web such as ciliates^15,22,38^. Therefore, diatoms likely increased the potential availability of food not only via high primary productivity but also via more accessible particle sizes^13,19,20,37^. Our findings suggest, however, that food quality may have been more important for the grazers than sheer quantity. Possibly a critical driver here were the extremely high carbon-to-nutrient ratios that occurred in association with diatoms across experiments and upwelling scenarios. This stoichiometric imbalance implies a shortage in vital nutrients such as N and P in grazers, leading to reduced secondary production^30,41^.

**Table 1:**
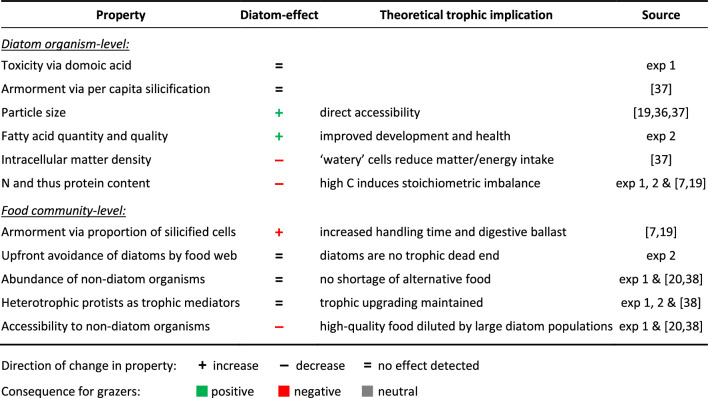
Possible pathways of diatom-driven impairment of copepod grazers during artificial upwelling in the oligotrophic ocean. Evidence ‘Source’ is restricted to this study (exp 1 and 2) and previous articles published on these experiments.

An abundance of non-diatom food biomass was maintained even under intense diatom blooms. Besides other autotrophs, this included a diverse assemblage of mixo- and heterotrophic dinoflagellates and ciliates (see this study and Spilling et al.^38^). Some of these protists are themselves prominent consumers of diatoms^25,42^ and can represent an upgrade in nutritional value^43^. Bypassing these trophic mediators via direct consumption of diatoms may hence not necessarily be advantageous for zooplankton or trophic efficiency^22,23^. This may help explain why in our study zooplankton did not shift their diet to the biomass-dominant diatoms and why food web length remained unchanged. Whilst zooplankton could have evaded low-quality diatoms via selective feeding, such behavioral strategies involve trade-offs^44^. The dilution and impeded accessibility of high-quality food via the disproportionate increase in diatoms could have contributed to the food web inefficiency.

**Figure 4:**
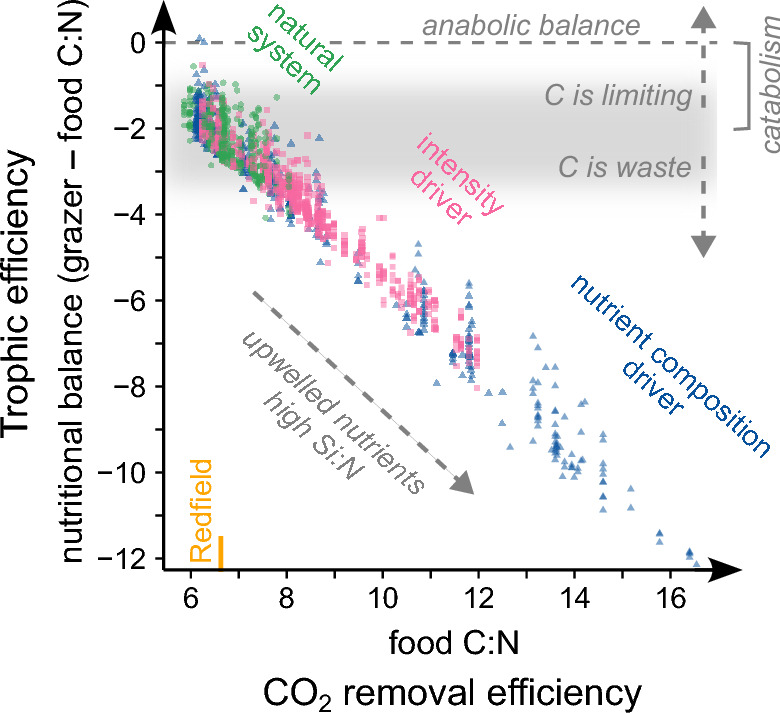
Potential conflict between food production and CO_2_ removal in microalgae-based artificial upwelling systems. Data shows herbivorous and omnivorous zooplankton (grazers) and particulate organic matter (potential food) across all experiments, mesocosms, time points and taxa. Overlay in grey conceptualizes the use of food carbon by the grazer.

Elemental stoichiometry not only drives food web processes but also biogeochemical cycles and therefore influences CO_2_ sequestration in addition to food production^45^. Both are ecosystem services of primary concern^2,46^ that artificial upwelling is considered to enhance. Our extensive dataset on ~ 1600 measures of suspended biomass and zooplankton stoichiometry, spanning various states of the pelagic system, may illustrate an intrinsic conflict between them (Fig. 4). Whenever the artificial system was pushed towards high CO_2_ uptake potential via carbon overconsumption in primary producers and C:N ratios beyond Redfield^11,45,47^, the mismatch between food and consumer stoichiometries grew. Whilst these are results from the sunlit surface, the high C:N ratios of exported particles observed during our experiments^7,10^ may similarly impact consumers of deeper ocean layers^48^. A net CO_2_ removal from the atmosphere may hence be inevitably linked to nutritionally imbalanced food webs with low fisheries production. Diatoms play a central role here, as in addition to driving trophic efficiency (whether positively or negatively), they are predestined for carbon overconsumption^29^ and a major contributor to the biological carbon pump^24,35^.

The strategies grazers adopt to cope with nutritional imbalances will shape the interdependency between ecosystem services. By only using carbon-rich molecules for energy, a hypothetical consumer could capitalize on resource C:N ratios multiple times higher than its own^41^, spanning almost the entire range observed during artificial upwelling (Fig. 4). Such perfect efficiency is unrealistic, though, and much of the excess carbon becomes costly waste^30^. To dispose of it, the consumer may either reduce its absorption during digestion, benefiting CO_2_ sequestration via elevated fecal pellet C:N ratios^34^, or excrete it post-absorption leading to a re-release of CO_2_^45,49^. The greatest advantage for trophic transfer, however, could come from a storage of the excess carbon in lipids that boosts reproduction and performance during periods of food limitation^50^. Stoichiometric flexibility is rare in consumers, though^40^, particularly in the oligotrophic surface ocean^51^, and we could not find evidence for it during artificial upwelling (Fig. 4).

A fertilization of microalgae in the open ocean typically results in highly dynamic food webs with a succession in biotic interactions and ecosystem services. Our mesocosms that operated at small spatiotemporal scales favored a mismatch situation between primary producers and zooplankton that may have amplified the impact of diatoms on trophic transfer. Based on observations from natural upwelling events in otherwise nutrient-poor waters^23^, simply employing larger scales may not improve trophic efficiency in response to blooming diatoms. Possibly, long periods of regular fertilization are required to undergo the necessary re-organization in zooplankton traits and species^52,53^. Such a state of ecological maturity may be achievable via a field of upwelling pumps that drift with the surface currents and continuously enrich the same patch of water.

A complete adaptation of grazer communities may generally not be expected if artificial upwelling is optimized for CO_2_ sequestration, as here carbon-to-nutrient ratios of microalgae are far beyond those of the natural system (Fig. 4, Ref.^47^). In contrast, other nature-based approaches including seaweed cultivation, restoration of coastal vegetation and afforestation^4,8,9,54^ operate already naturally at high carbon-to-nutrient ratios allowing for a high degree of specialization in grazers. In these systems, CO_2_ removal and trophic transfer can also be to some extent decoupled when carbon storage is provided by large structures that are low in nutritional value but important as habitat for biodiversity (e.g. macrophytes, wood) and food by smaller and more palatable structures (e.g. epiphytes, leaves). Artificial upwelling in combination with seaweed cultivation^55^ may hence alleviate the trade-off between food production and CO_2_ removal.

Our finding of diatom-driven trophic inefficiency contrasts the classical food web model^12,15^ and would thus reduce the fisheries potential of artificial upwelling. Food quality can be more critical than quantity to allow for fast population growth of metazoan zooplankton during shorter-term fertilization of the oligotrophic ocean. Under these circumstances, application strategies of artificial upwelling that restrain the proliferation of diatoms, including low silicate in source water and more moderate levels of nutrient enrichment, may be preferred when the goal is to maximize trophic efficiency. Similar food web dynamics could emerge during natural upwelling events in otherwise oligotrophic waters created via mesoscale eddies or fronts. The global decline in diatoms in a future ocean^35,56^ may hence not signify reduced fish production everywhere. We further describe an intrinsic conflict between the ecosystem services of food production and CO_2_ removal underlying the carbon-to-nutrient ratio of biological processes. By presenting the special case of a highly dynamic system with microscopic primary producers, our study contributes to the evaluation of nature-based solutions for climate change mitigation and food security.

## Methods

### Study system

Experiments were conducted off Gran Canaria, an island surrounded by deep subtropical ocean. Here, resident plankton communities are characteristic of warm, nutrient-poor waters^15^. Nitrate is limiting phytoplankton productivity^19,20,39^, which is dominated by smaller taxa including cyanobacteria, haptophytes, prasinophytes and chlorophytes^20,36,37^. A diverse assemblage of diatoms is also present, albeit at low abundance, including common genera like *Leptocylindrus*, *Pseudo-nitzschia*, *Guinardia* and *Chaetoceros*^19,20,57^. Mixo- and heterotrophic dinoflagellates and ciliates are key trophic intermediaries (Fig. S3d,e, Ref.^38^). Meso-zooplankton grazers are dominated by copepods with *Temora*, *Paracalanus*, *Nannocalanus*, *Centropages*, *Oithona* and *Oncaea* being amongst the most common (Figs. S2a, S5a)^58,59^. These small to medium-sized genera are considered important prey for small pelagic fishes and fish larvae^59^. We enclosed the local plankton community for several weeks in mesocosm consisting of 2-m wide, transparent plastic bags with cylindrical sediment trap.

### Nutrient composition: experiment 1

Eight smaller mesocosms were maintained inside Taliarte harbor (27°59′24″ N, 15°22′8″ W) for 33 days from September to October 2019. These units had a volume of ~ 8.3 m^3^ with a depth of ~ 3.5 m. Mesocosms were filled (day 0) with seawater from outside the harbor using a peristaltic pump, while a 3 mm mesh excluded larger organisms. Throughout the experimental period, the shallow water column remained well mixed with a temperature of 22.2–23.2 °C, salinity of ~ 36.5 and O_2_ close or above saturation with 215–320 µmol kg^−1^. Photosynthetically active radiation (PAR) decreased from ~ 600 to ~ 300 µmol m^−2^ s^−1^ from the surface to the sediment trap. Further details about the experimental setup, abiotic environment^19^, phytoplankton community^37^ and particle sedimentation^10^ is provided in preceding articles.

Across these mesocosm units, we established a gradient in silicate relative to nitrate (*Si:N*) ranging from extreme Si deficiency (0.07) to excess Si (1.33) (see Fig. 2 in Goldenberg et al.^19^). Silicate is co-limiting the growth of diatoms but not of other primary producers^24^. Thereby, a ratio of silicate to nitrate of 1:1 or above is considered optimal for diatoms, yet with considerable variability between and within species. For treatment application, deep water with 30 µmol L^-1^ nitrate was prepared by supplementing subsurface water (~ 140 m depth) with macro-nutrients in Redfield proportions, except silicate which varied for each mesocosm. Regular upwelling was simulated by replacing 4% of the mesocosm volume with deep water every second day, starting on day 6. By manipulating silicate under otherwise identical upwelling conditions, this experiment represents a more direct test for diatoms and is thus introduced first. We expected an increased dominance of diatoms in phytoplankton from low to high Si availability.

Small pelagic fish form the center of upwelling food webs by transferring energy from zooplankton to fisheries^17^. Following 8 days of upwelling, our food webs were complemented with the locally caught silverside *Atherina presbyter*. Each mesocosm received 45 young juveniles (mean ± SD total length = 17.2 ± 1.2 mm; wet mass = 20.9 ± 4.6 mg) and 36 larvae (total length = 9.0 ± 1.2 mm; wet mass = 2.9 ± 1.4 mg) that overlap in trophic function with key fisheries species such as sardine and anchovy (Fig. S1a). After 6 days, the fish had depleted the zooplankton and were removed with a net of 1 mm mesh spanning the width of the mesocosm. This final fish biomass (day 21) was used in the analysis as indicator of fish performance. Whilst on average 44 out of 45 juveniles could be recovered, the more sensitive larvae showed high and random mortality (Fig. S1b). We thus based our investigation on the juveniles only, representing 93% of fish biomass. To assess fish feeding, a subset of ~ 7 juveniles had been caught from each mesocosm on day 18 before zooplankton depletion. Prey organisms in stomachs were counted and photographed using a stereo microscope to estimate prey biovolume and ultimately carbon mass. Due to right-skewedness, the derived feeding success variable was log_10_-transformed at the level of individual fish. The removal of the fish marked the end of the multi-level food web and hence this study. The experiment continued for another 2 weeks with the simplified community of phytoplankton and heterotrophic protist.

The base of the food web was monitored in regular intervals throughout the experiment. Depth-integrated water samples were taken from the pier via plastic tubes (Ø 53 mm, 2.5 m, 5.1 L). Water was filtered (> 0.7 µm) for particulate organic matter (POM) C (POC), N (PON) and P (POP), and photosynthetic pigments including chlorophyll *a* (Chl *a*) in 2-day intervals and toxins in 4-day intervals. Pigments were analyzed via reverse-phase high-performance liquid chromatography (HPLC) and used to estimate phytoplankton community composition with Chemtax v.1.95 based on Higgins et al.^60^ (RMS = 0.024). As the plankton community contained only *Pseudo-nitzschia* as a genus capable of producing known phycotoxins, namely domoic acid and its variants^27^, we analysed for this toxin following Krock et al.^61^. Particulate matter filtrates were extracted with methanol, adjusted to a final volume of 300 µL and analyzed by liquid chromatography coupled to tandem mass spectrometry (LC–MS/MS) in the selected reaction monitoring (SRM) mode using positive ionization. The biovolume of diatoms and heterotrophic protists was assessed by Utermöhl light microscopy in 2-day intervals. Heterotrophic protists were further converted to carbon and nitrogen^62,63^. We restricted the data analysis for nutritional value (N content and toxins) and non-diatom food options (auto- and heterotrophs) to before fish introduction. This was done to isolate the period of bottom up control on zooplankton, during which the zooplankton populations diverged according to the upwelling treatment. The full dataset is provided in the supplement (Fig. S3).

In our study, ‘zooplankton’ referred to all metazoan zooplankton larger than 55 µm including larvae, juveniles and adults. For all parameters, samples were first split into three size fractions: 55–200, 200–500 and > 500 µm. Abundance was assessed throughout the experiment in 2-day intervals based on triplicate tube samples (Ø 53 mm, 2.5 m, 5.1 L). Organisms were identified and counted under a stereo microscope following preservation with 70% ethanol. In both experiments, the crustacean zooplankton primarily comprised small- and medium-sized copepod species (Figs. S2a, S5a), which served as model grazers in our study. For isotope trophic markers and C and N content, organisms were caught with nets (Apstein Ø 17 cm, 55 μm mesh) and picked fresh into tin capsules in groups (> 5 µg C/sample), oven-dried at 60 °C and measured in an element analyser coupled to a mass spectrometer. We employed the trophic position proxy δ^15^N that is enriched by ~ 2 ‰ with each trophic step^64^. Trophic markers in zooplankton integrate lower-level processes over several days and hence respond with a time delay. Day 13 was considered for the data analysis of trophic position, being the only day available after upwelling treatment and before fish feeding. To obtain a primary producer baseline, the POM δ^15^N sampled over the three days preceding the respective zooplankton sample was averaged. This baseline was corrected for its heterotrophic fraction of protists and zooplankton.

### Upwelling intensity: experiment 2

Nine large mesocosms were studied in a sheltered bay not far from the first experiment (27°55′40″ N, 15°21′52″ W) for 38 days from November till December 2018. They had a volume of ~ 43 m^3^ with a depth of ~ 15 m. The lower end of the mesocosm bags was extended to depth, while open at the bottom, to enclose a column of seawater. The bottom was then closed (day 0) to isolate the bags from the surrounding Atlantic. Larger organisms were excluded using a net with a mesh of 3 mm that spanned the width of the mesocosms. Despite its larger depth, the water column remained well mixed throughout the experimental period. This was due to continuous temperature equilibration with the unstratified coastal current passing by the mesocosms. Temperature was between 20.7 and 21.6 °C, salinity at ~ 36.9 and O_2_ close or above saturation with 207–314 µmol kg^−1^. Photosynthetically active radiation (PAR) ranged from ~ 520 to ~ 80 µmol m^−2^ s^−1^ from the surface to the sediment trap. Overall, abiotic conditions were similar in the two experiments, except for the lower irradiance at depth in the larger mesocosms. Fish larvae (*Diplodus sargus*) were introduced also in this experiment but did not survive. Zooplankton were hence the highest trophic level tested here. Further details about the experimental procedures, phytoplankton communities^20,36^, heterotrophic protists^38^ and particle sedimentation^7^ are provided in preceding articles.

We tested the amount (*intensity*) and duration (*mode*) of nutrient upwelling (see Table 1 in Baumann et al.^7^). For this, we established a 5-step gradient in *intensity* ranging from 0 to ~ 10 µmol L^−1^ nitrogen. This treatment variable represented the cumulative N enrichment across all N pools (dissolved inorganic, dissolved organic and particulate organic). Starting on day 4, deep water was added to half of the mesocosms in 4-day intervals (*recurring mode*), while the other half received all their deep water in one large addition (*singular mode*). With this, we mimicked two implementation strategies: a free-floating facility that enriches a water body continuously (‘patch’ type fertilization) versus a stationary facility that supplies only a single pulse of nutrients before the water is swept away by currents (‘plume’ type fertilization). An extra mesocosm served as no-upwelling *control*. For the upwelling manipulation, deep water of 25 µmol L^−1^ nitrate was prepared by supplementing subsurface water (~ 300 m depth) with macro-nutrients in Redfield proportions. Si:N was maintained at a constant, intermediate level of 0.8^24^. We expected an increase in diatom dominance with upwelling *intensity*, and, for the *singular mode*, a decrease from bloom to post-bloom conditions.

The base of the food web was monitored again in regular intervals. Depth-integrated sampling was performed from boats using automated water samplers (5 L). POC, PON, POP and Chl *a* were assessed in 2-day intervals as in the first experiment. Fatty acids in POM (stored at – 80 °C) were measured in 4-day intervals by gas chromatography according to Dorner et al.^65^. The biovolume of diatoms and heterotrophic protists was assessed by flow imaging analysis with a FlowCam (Fluid Imaging)^38^. Heterotrophic protists were further converted to carbon and nitrogen^62,63^. For all variables, the entire upwelling period was used in the data analysis given that this experiment was not divided by the presence/absence of fish.

Zooplankton was sampled with nets (Apstein Ø 17 cm with 55 μm mesh; Ø 50 cm with 500 µm mesh) and split into size-fractions. Abundance was assessed in 8-day intervals via microscopy (Fig. S5a). The trophic marker δ^15^N and C and N content were measured in 4-day intervals. For analysis of δ^15^N, only samples from day 11 onwards were used to consider time for trophic marker incorporation into zooplankton tissue. This matched the time lag employed in the first experiment. Fatty acid trophic markers were instead only sampled two times at the end (day 30 and 36) because of logistical constraints. For this, organisms were briefly thawed for transfer into tin capsules in groups (180 µg C/sample on average), freeze dried and then analysed like the filters. The marker 20:5ω3/22:6ω3 (EPA/DHA) was most suitable to track the propagation of diatom productivity up the food web^20,28,50^. Marker strength in copepod samples was compared to that in POM averaged over the 10 preceding days.

### Data analysis

Food web responses were tested with linear regressions. The upwelling manipulation *Si:N* (exp 1) and *intensity* (exp 2) and the emerging system property of diatom dominance (exp 2) were employed as continuous explanatory variables. The latter represented the relative contribution of diatoms to the phytoplankton community, estimated as the ratio of diatom biovolume to total chlorophyll *a*. Repeated measures of both explanatory and response variables were averaged for the period of interest to obtain one value per mesocosm. Under this temporal integration, upwelling *mode* was only a weak diatom driver and excluded from the main analyses (Table S2).

The more general investigation into ecological stoichiometry was instead conducted at the level of individual sampling days and across experiments and treatments. All available samples were included. Grazers were correlated with particulate organic matter that had been averaged over the 3 preceding days. To regard for the repeated measure, we employed linear mixed models with *mesocosm* as random effect. The oligotrophic phase before deep water addition as well as all sampling days of the *control* mesocosm and the *Atlantic* water surrounding the facility represented the *natural* system state.

We performed all analyses at a significance level of α = 0.05 with R version 4.0.5^66^. Normality of residuals was checked with normal Q-Q plots and homogeneity of variance with residual versus fitted plots. Data was transformed if necessary.

### Ethics

Animal research was approved under OEBA -ULPGC 12/2019R1 and OEBA-ULPGC-13/2018 and the collection of wild fish under (1036086, AGPA 62777, 28/06/2019) by the Government of the Canary Islands. All experiments followed the relevant guidelines and regulations.

### Supplementary Information

Supplementary Information.

## Data Availability

The raw data supporting the conclusions of this article are available at 10.1594/PANGAEA.954852, 10.1594/PANGAEA.963781, 10.1594/PANGAEA.963468, 10.1594/PANGAEA.963462, 10.1594/PANGAEA.963467, 10.1594/PANGAEA.951417 (experiment 1) and 10.1594/PANGAEA.963590, 10.1594/PANGAEA.963541, 10.1594/PANGAEA.963589 (experiment 2).
