# Supplementary material for: Diatom-mediated food web functioning under ocean artificial upwelling
Source: Sci Rep. 2024 Feb 17;14:3955. doi: 10.1038/s41598-024-54345-w (PMC10874431; doi:10.1038/s41598-024-54345-w)
Supplement: Supplementary file 1 — Supplementary Information. [file 41598_2024_54345_MOESM1_ESM.pdf]

Supplementary information

Goldenberg et al - Diatom-mediated food web functioning under ocean artificial upwelling

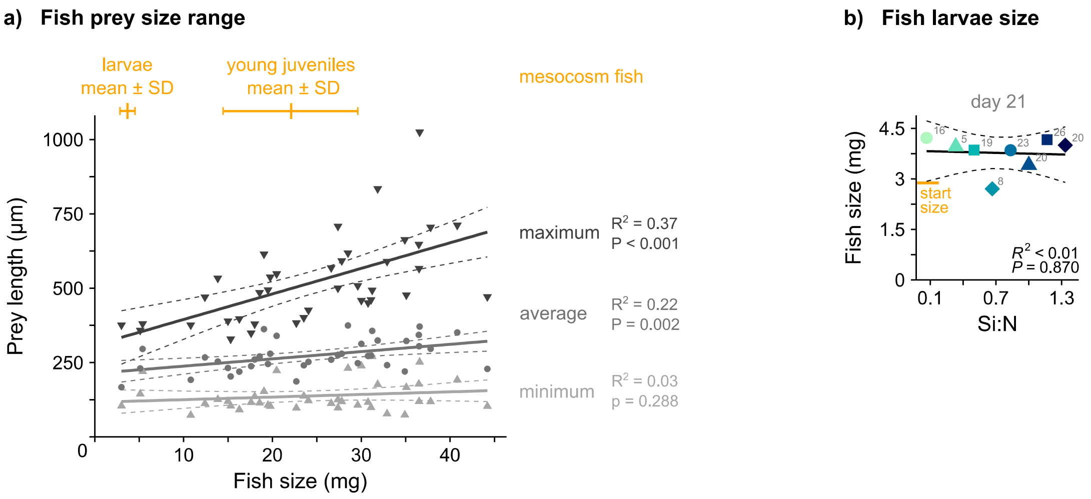

Figure S1: Fish of the nutrient composition experiment. a) Natural prey size range of the study fish. Individuals that had been feeding on the wild plankton community in the harbor were analyzed for stomach content. The longest dimension of the prey body (prosome for copepods) was assessed under a stereomicroscope. For each fish individual, the smallest and largest prey found and the average size across all prey are shown and employed in linear regressions (± 95% confidence ranges). The size reference of the fish used for the experiments inside the mesocosms is provided in orange. Our fish overlap strongly in prey size range, and thus trophic function, with both larvae and adults of commercially important small pelagic fishes such as sardines and anchovies [1-4]. **b)** Size of fish individuals of the larvae class after 6 days inside the mesocosms under Si:N manipulation. Averages across several individuals (# in grey) were employed in linear regressions. Abundances were highly variable, assumable due to random mortality of this more sensitive life stage, and unrelated to Si:N (regression: $R^2 = 0.25$, $p = 0.206$).

Table S1: Linear regressions of the nutrient composition experiment with Si:N as explanatory variable to accompany fig 1.

Response variable	Source of variation	df	MS	F-ratio	p	R^2
**a)** Diatom biovolume (mean days 9-21)	Si:N	1	257	36.95	<0.0001	0.86
	Residuals	6	7			
**b)** Nauplii abundance (mean days 9-21)	Si:N	1	38.1	36.11	<0.0001	0.86
	Residuals	6	1.1			
**c)** Copepod mass (mean days 9-21)	Si:N	1	1.47	12.67	0.0120	0.68
	Residuals	6	0.12			
**d)** Copepod mass in fish gut (day 18)	Si:N	1	0.698	24.69	0.0025	0.80
log10-transformed	Residuals	6	0.028			
**e)** Fish mass juveniles (day 21)	Si:N	1	24.1	8.70	0.0257	0.59
	Residuals	6	2.8			
**f)** $\delta^{15}N$ copepods – autotroph baseline (day 13)	Si:N	1	0.259	0.81	0.4036	0.12
	Residuals	6	0.321			
**g)** Particulate organic N:C (mean days 9-15)	Si:N	1	0.00238	20.38	0.0040	0.77
	Residuals	6	0.00012			
**h)** Domoic acid per POC (mean days 9-15)	Si:N	1	32.8	0.64	0.4554	0.10
	Residuals	6	51.6			
**i)** Non-diatom chlorophyll a (mean days 9-15)	Si:N	1	0.0069	0.08	0.7832	0.01
	Residuals	6	0.0829			
**j)** Heterotrophic protists (mean days 9-15)	Si:N	1	0.26	0.06	0.8200	<0.01
	Residuals	6	4.65			

df = degrees of freedom; MS = mean squares

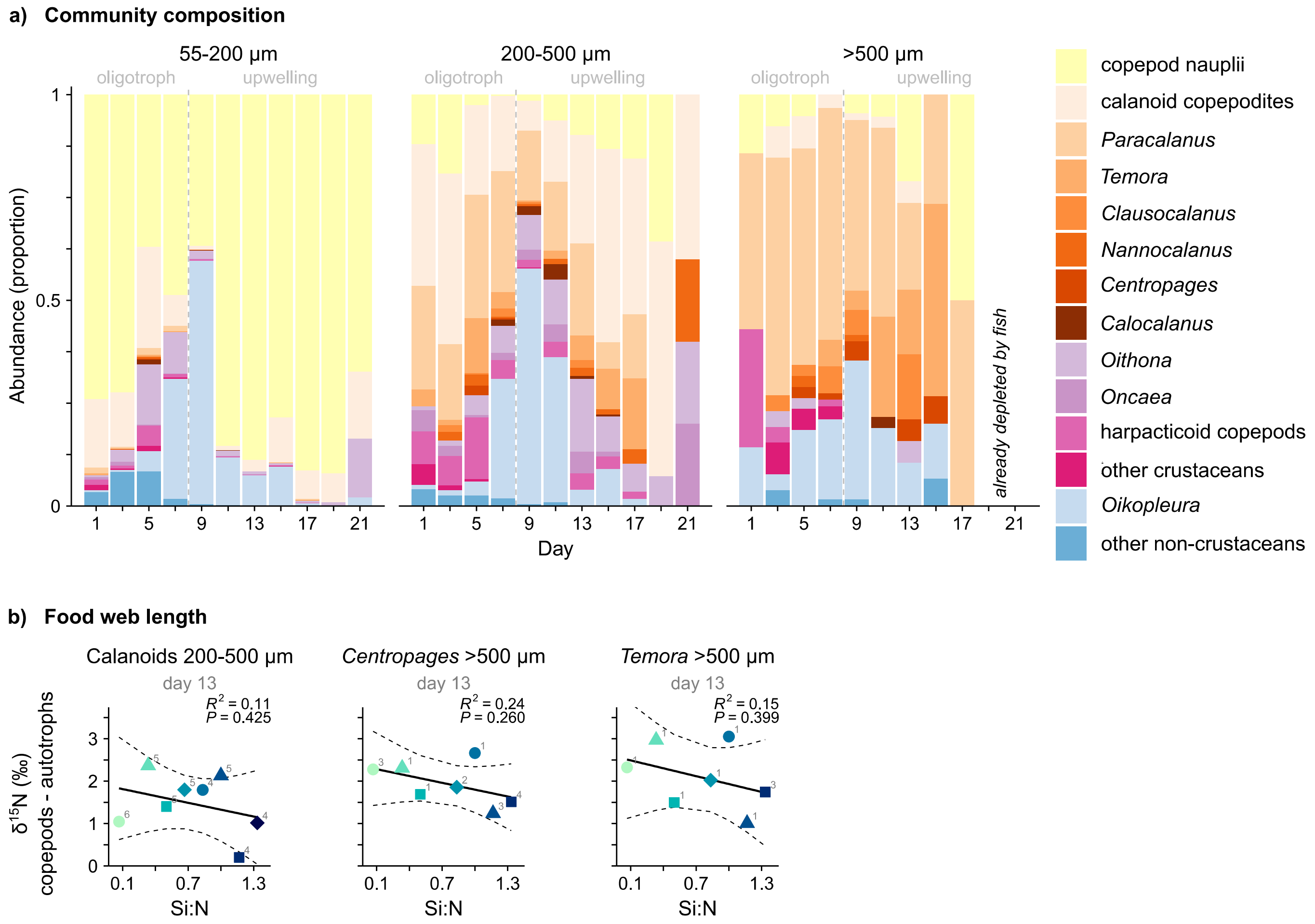

Figure S2: Zooplankton during the nutrient composition experiment. a) Abundance-based community composition including all metazoan zooplankton averaged across all mesocosms for each size class. Note that the high *Oikopleura* counts on day 9 come from a short and randomly occurring bloom of these organisms in a single mesocosm. The copepods comprise mainly herbivorous and omnivorous taxa that are common in the oligotrophic surface ocean [5] and important prey for small pelagic fishes and fish larvae of other fisheries species [6,1,2,7,3,4]. **b)** Trophic level estimate for different grazers, expressed as the enrichment in $d^{15}N$ in copepods compared to the phytoplankton baseline. Averages across several samples (# in grey) were employed in linear regressions (± 95% confidence ranges).

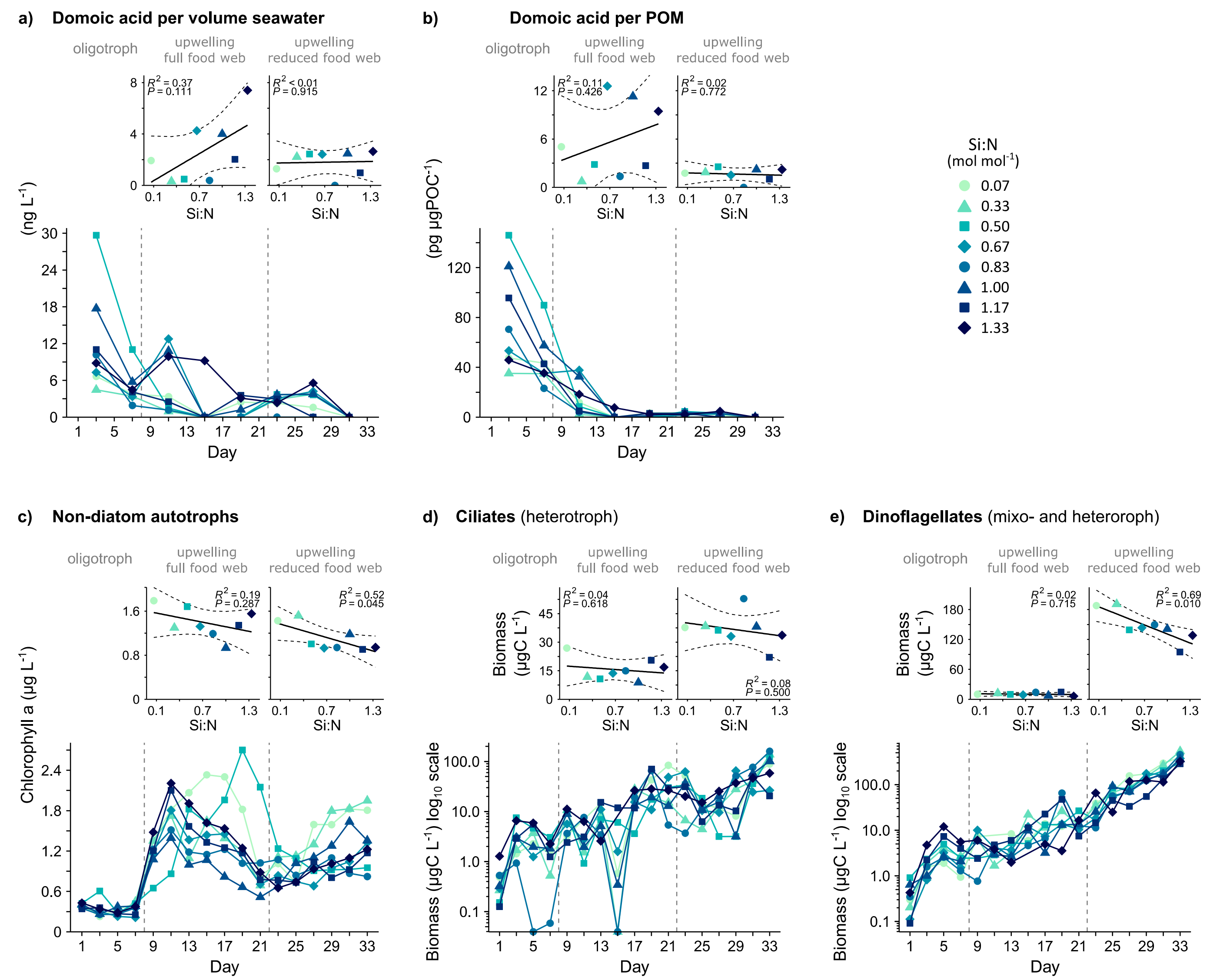

Figure S3: Food web base during the nutrient composition experiment. Shown are temporal developments and phase averages employed in regressions (95% confidence ranges via dashed lines).

Table S2: Linear models to establish that, integrated over the experimental period of the upwelling intensity experiment, upwelling *mode* was only minor driver of diatoms **(a)** and fatty acids **(b)**. *Intensity* (continuous), *mode* (categorical) and their interaction were employed as explanatory variables (type III test).

Response variable	Source of variation	MS	df	F-ratio	p
**a)** Diatom biovolume (mean days 7-37)	Intensity	14.8	1	107.35	0.0005
	Mode	0.7	1	5.17	0.0853
	Intensity × Mode	0.2	1	1.56	0.2800
	Residuals	0.1	4		
**b)** Total fatty acids (mean days 7-37)	Intensity	532133	1	425.34	<0.0001
	Mode	744	1	0.59	0.4836
	Intensity × Mode	9885	1	7.90	0.0483
	Residuals	1251	4		

MS = mean squares; df = degrees of freedom

Table S3: Linear regressions for the upwelling intensity experiment to accompany figure 2.

Response variable	Explanatory variable	Source of variation	df	MS	F-ratio	p	R^2
**a)** Diatom biovolume (mean days 7-37)	Upwelling intensity	Intensity	1	18.9	85.18	<0.0001	0.92
		Residuals	7	0.2			
**b)** Diatom marker in copepods (mean days 30-36)	Diatom marker in POM	Intensity	1	0.0284	21.99	0.0034	0.79
		Residuals	7	0.0013			
**c)** $\delta^{15}N$ copepods - autotrophs (mean days 11-38)	Diatom dominance	Intensity	1	0.012	0.04	0.8455	<0.01
		Residuals	7	0.286			
**d)** Total fatty acids (mean days 7-37)	Upwelling intensity	Intensity	1	599946	133.45	<0.0001	0.95
		Residuals	7	4496			
**e)** % ARA + DHA + EPA (mean days 7-37)	Diatom dominance	Intensity	1	75.3	47.36	0.0002	0.87
		Residuals	7	1.6			
**f)** Particulate organic N:C (mean days 7-37)	Diatom dominance	Intensity	1	0.000766	18.23	0.0037	0.72
		Residuals	7	0.000042			

df = degrees of freedom; MS = mean squares

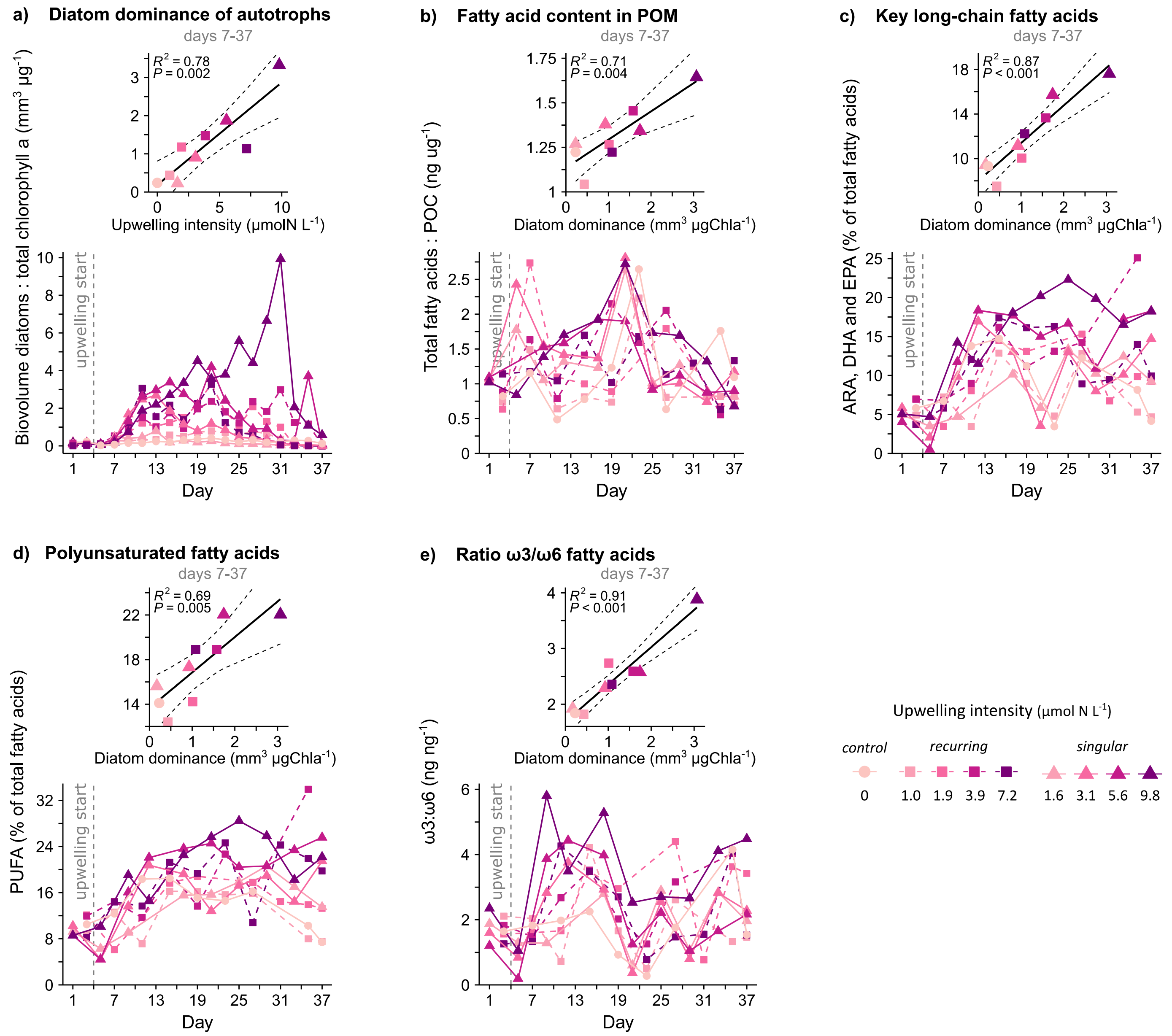

Figure S4: Food web base during the upwelling intensity experiment. **a)** Full illustration of 'diatom dominance' as the explanatory variable used in several further analyses. **b-e)** Particulate organic matter fatty acids. Shown are temporal developments and phase averages employed in regressions (95% confidence ranges via dashed lines).

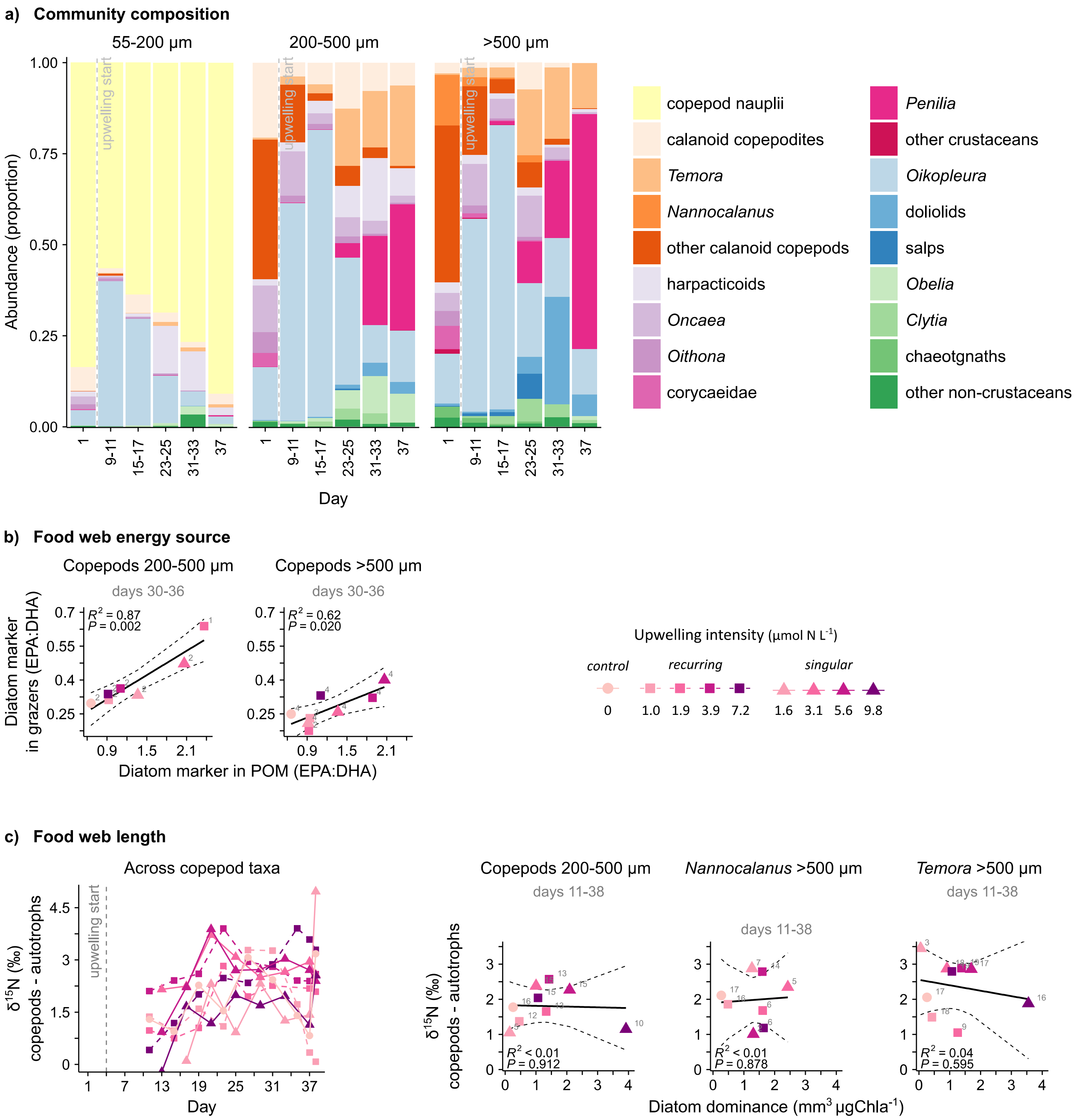

Figure S5: Zooplankton during the upwelling intensity experiment. a) Abundance-based community composition including all metazoan zooplankton averaged across all mesocosms for each size class. Note that while Oikopleura has high abundances, its body is partly gelatinous with low organic matter content and its long tail makes it collect in larger size classes. In terms of biomass, the composition pattern would thus shift in favor of the copepods. **b)** Fatty acid - based estimate on the change in the the contribution of diatoms to the diet of copepods. All samples of the two size classes are combined for figure 2b. **c)** Trophic level estimate for grazers expressed as the enrichment in d^{15}N in copepods compared to the phytoplankton baseline: across groups per day (left) and across days per group (right). The three groups were combined for figure 2c. Averages across several samples (# in grey) were employed in linear regressions (± 95% confidence ranges).

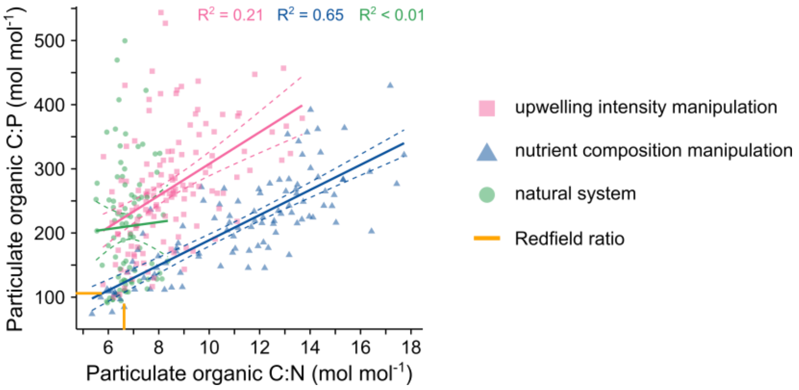

Figure S6: Relationship between P and N content in particulate organic matter during artificial upwelling. All sampling days of both experiments are included as individual data points and employed in regressions (95% confidence ranges via dashed lines). Three data points with extremely high C:P ratios were seen as technical errors and excluded.

Table S4: Linear mixed models for the relationship between zooplankton and particulate organic matter stoichiometry. Both experiments were included with one data point per mesocosms and sampling day. Particulate organic C:N was employed as continuous fixed effect and *mesocosm* (n=18) as random effect (random intercept, restricted maximum likelihood fit, Satterthwaite approximation, [8,9]).

Response variable	MS	df_{Num}	df_{Den}	F-ratio	p
**a)** Crustaceans <200 µm C:N	1.277	1	112	5.34	0.0226
**b)** Crustaceans 200-500 µm C:N	0.188	1	145	1.43	0.2334
**c)** Crustaceans >500 µm C:N	0.117	1	113	1.75	0.1881
**d)** Tunicates >55 µm C:N	0.014	1	51	0.102	0.7502

MS = mean squares; df_{Num} and df_{Den} = numerator and denominator degrees of freedom

References:

1. Plounevez S and Champalbert G (2000) Diet, feeding behaviour and trophic activity of the anchovy (*Engraulis encrasicolus* L.) in the Gulf of Lions (Mediterranean Sea). *Oceanol. Acta*, 23:175-192.
2. Van der Lingen CD (2002) Diet of sardine *Sardinops sagax* in the southern Benguela upwelling ecosystem. *South Afr. J. Mar. Sci.-Suid-Afr. Tydsk. Seewetens.*, 24:301-316.
3. Borme D, Tirelli V, Brandt SB et al. (2009) Diet of *Engraulis encrasicolus* in the northern Adriatic Sea (Mediterranean): ontogenetic changes and feeding selectivity. *Mar. Ecol.-Prog. Ser.*, 392:193-209.
4. Morote E, Olivar MP, Villate F et al. (2010) A comparison of anchovy (*Engraulis encrasicolus*) and sardine (*Sardina pilchardus*) larvae feeding in the Northwest Mediterranean: influence of prey availability and ontogeny. *ICES J. Mar. Sci.*, 67:897-908.
5. Hernandez-Leon S (1998) Annual cycle of epiplanktonic copepods in Canary Island waters. *Fish Oceanogr.*, 7:252-257.
6. Turner JT (1984) The feeding ecology of some zooplankters that are important prey items of larval fish. *NOAA Technical Report NMFS 7*.
7. Turner JT (2004) The importance of small planktonic copepods and their roles in pelagic marine food webs. *Zool. Stud.*, 43:255-266.
8. Bates D, Machler M, Bolker BM et al. (2015) Fitting Linear Mixed-Effects Models Using lme4. *J. Stat. Softw.*, 67:1-48.
9. Kuznetsova A, Brockhoff PB and Christensen RHB (2017) lmerTest Package: Tests in Linear Mixed Effects Models. *J. Stat. Softw.*, 82:1-26.
